# The correlation between serum HBsAg levels and viral loads depends upon wild‐type and mutated HBV sequences rather than the HBeAg/anti‐HBe status

**DOI:** 10.1002/jmv.24186

**Published:** 2015-04-16

**Authors:** Mo‐Han Liu, Qin‐Yan Chen, Tim J. Harrison, Guo‐Jian Li, Hai Li, Xue‐Yan Wang, Yu Ju, Jin‐Ye Yang, Zhong‐Liao Fang

**Affiliations:** ^1^ Department of MicrobiologySchool of Preclinical MedicineGuangxi Medical UniversityNanningGuangxiChina; ^2^ Guangxi Zhuang Autonomous Region Center for Disease Prevention and ControlNanningGuangxiChina; ^3^ Guangxi Key Laboratory for the Prevention and Control of Viral HepatitisNanningGuangxiChina; ^4^ Division of MedicineUCL Medical SchoolLondonUK; ^5^ Department of Public Health of Guangxi Zhuang Autonomous RegionNanningGuangxiChina

**Keywords:** hepatitis B virus, HBsAg, mutations, correlation, viral loads

## Abstract

Despite several studies regarding the correlation between serum HBsAg titers and viral loads, the association remains uncertain. Eighty‐nine individuals were selected randomly from a Chinese cohort of 2,258 subjects infected persistently with hepatitis B virus (HBV) for cross‐sectional and longitudinal analysis. Viral loads of mutant HBV are lower than those of wild type HBV. The serum HBsAg titers correlate positively with viral loads in both HBeAg positive and negative subjects (r = 0.449, *P* = 0.013; r = 0.300, *P* = 0.018, respectively). No correlation between serum HBsAg titer and viral loads was found in any of the four phases of chronic HBV infection. The serum HBsAg titers correlate positively with viral loads in the group with wild type sequences of the PreS/S, basal core promoter (BCP), and preC regions of HBV(r = 0.502, *P* = 0.040). However, the correlation was not seen in the group with mutations in these regions (r = 0.165, *P* = 0.257). The correlation between HBsAg titers and viral loads was seen in individuals with wild type PreS/S sequences but not in the subgroup with BCP double mutations or PreC stop mutation, although their sequences in the preS/S regions were wild type. All these findings were confirmed by the longitudinal analysis. In conclusion, the correlation between serum HBsAg levels and viral loads may not differ between HBeAg positive and negative individuals but may depend on wild‐type or mutated genomic sequences. Therefore, HBsAg quantitation may be used as a surrogate for viral loads in only wild‐type HBV infections. ***J. Med. Virol. 87:1351–1360, 2015*.** © 2015 The Authors. *Journal of Medical Virology* Published by Wiley Periodicals, Inc.

## INTRODUCTION

Hepatitis B virus (HBV) has a circular, partially double‐stranded DNA genome of about 3200 nucleotides with four open reading frames (ORFs), namely the precore/core, polymerase, surface, and X ORFs [Tiollais et al., 1985]. Monitoring the serum concentrations of HBV DNA has been used to predict the outcome of chronic HBV infection and the response to antiviral therapy [Yuen et al., 2007; Yuen et al., 2009]. However, HBV DNA quantification remains expensive and, more importantly, it is not easily accessible or affordable in developing counties where HBV infection is prevalent [Su et al., 2010].

Hepatitis B surface antigen (HBsAg) was the first HBV protein discovered in 1965 [Blumberg et al., 1965]. The HBsAg found in serum can include all three forms of the surface protein found in the mature HBV virion, the large (L), medium (M), and small (S) proteins. These proteins are encoded by the S‐ORF which contains three, in‐frame initiation codons and is divided into the Pre‐S1, Pre‐S2 and S domains [Locarnini and Bowden, 2012]. Over the years, detection of HBsAg in serum has been the hallmark of HBV infection and remains the cornerstone for the diagnosis of acute and chronic hepatitis B [Martinot‐Peignoux et al., 2014]. HBsAg quantification has been recognized as valuable for monitoring the natural history of chronic hepatitis and predicting treatment outcome [Martinot‐Peignoux et al., 2013]. Compared to HBV DNA, quantification of HBsAg is relatively inexpensive and has been used widely.

Since the first report in 2004 of a positive correlation between the serum HBsAg titer and HBV DNA concentrations [Deguchi et al., 2004], several studies have yielded similar results [Gupta et al., 2012; Alghamdi et al., 2013; Suh et al., 2014] and suggested that the serum HBsAg titer may be used as a surrogate marker of serum HBV DNA in chronic HBV infection [Su et al., 2010; Sun et al., 2012]. However, at the same time, there are some contradicting results from other studies suggesting that quantitative HBsAg assays cannot substitute for HBV DNA quantification [Kuhns et al., 2004; Wiegand et al., 2008; Ganji et al., 2011]. Although a further study suggested that the correlation between quantitative HBsAg titers and serum HBV DNA differs between HBeAg‐positive and HBeAg‐negative patients with chronic hepatitis B (CHB) [Thompson et al., 2010], the contradictory results remain unexplained. Some studies found the correlation in HBeAg‐positive CHB patients [Su et al., 2010, 2012; Suh et al., 2014] while others found the correlation in HBeAg‐negative CHB patients [Seto et al., 2012; Alghamdi et al., 2013].

The high rate of viral replication, combined with an error‐prone polymerase, results in a very high frequency of mutational events during HBV infection. The most commonly mutations are the preC stop mutation (G1896A) that prevents the synthesis of HBeAg, BCP double mutations (A1762T, G1764A), and PreS mutations [Harrison, 2006]. It has been reported that BCP double mutations and preS mutations have an impact on HBV replication [Buckwold et al., 1996; Bock et al., 1997; Kondo et al., 2002]. Recently, a cross‐sectional analysis showed that the correlation between serum HBsAg titers and HBV DNA concentrations differs between wild type and mutated preS/S sequences. However, the authors did not report an analysis according to the sequence status of the BCP and preC [Pollicino et al., 2012].

In this study, a cross‐sectional analysis was carried out to determine the correlation between serum HBsAg titers and HBV DNA concentrations, according to the sequence status of HBV, and included a longitudinal analysis to test this correlation further.

## MATERIALS AND METHODS

### Study Subjects and Sample Design

The study subjects were recruited from the Long An cohort, which has been described previously [Fang et al., 2008]. The cohort was recruited in early 2004 from agricultural workers aged 30–55 living in the rural area of Long An county, Guangxi, China, using stratified sampling. We started to follow up the study subjects from 1st July, 2004. Each study subject provided a serum sample every six months for the assessment of virological parameters and AFP concentrations. In this study, the study subjects were selected from the first to third round follow‐up according to the availability of serum for quantification of HBsAg, measurement of viral loads and HBV DNA sequencing, and their HBeAg/anti‐HBe status was stable (at least for the period covering the three tests).

Informed consent in writing was obtained from each individual. The study protocol conforms to the ethical guidelines of the 1975 Declaration of Helsinki and has been approved by the Guangxi Institutional Review Board.

### Serological Testing

Sera were tested for HBsAg and HBeAg/anti‐HBe using enzyme immunoassays (Zhong Shan Biological Technology Company, Limited, Guangzhou, China). Alanine aminotransferase (ALT) levels were determined using a Reitman kit (Sichuan Mike Scientific Technology Company, Limited, Chengdu, China).

### HBsAg Measurement

Serum HBsAg titers were measured by a commercial chemiluminescent microparticle immunoassay using the ARCHITECT i2000SR platform and Abbott Architect HBsAg reagents (Abbott Ireland Diagnostics Division, Finisklin Business Park, Sligo, Ireland) with a dynamic range of 0.05‐250 IU/ml. In cases where the HBsAg titers were >250 IU/ml, serial 1:500 dilutions were performed according to the manufacturer's instructions.

### Nested PCR for HBV DNA and Nucleotide Sequencing

DNA was extracted from 85 µl serum by pronase digestion followed by phenol/chloroform extraction. To amplify the preS/S region, the first round polymerase chain reaction (PCR) was carried out in a 50 µl reaction using primers LSOB1 (nt 2739‐2762, 5′‐GGCATTATTTGCATACCCTTTGG‐3′) and MDN5R (nt1794‐1774, 5′‐ATTTATGCCTACAGCCTCCT‐3′) with 5 min hot start, followed by 30 cycles of 94^°^C for 30 sec, 50^°^C for 30 sec, and 72^°^C for 90 sec. Second round PCR was carried out on 5 µl of the first round products in a 50 µl reaction using primers LSBI1 (nt 2809‐2829, 5′‐TTGTGGGTCACCATATTCTT‐3′) and HCO2 (nt 761‐776, 5′‐GCGAAGCTTGCTGTACAGACTTGG‐ 3′) and the same amplification protocol as first round.

To amplify the preC/BCP region, the first round PCR was carried out in a 50 µl reaction using primers B935 (nt 1240–1260, 5′‐AACCTTTGTGGCTCCTCTG‐3′) and MDC1 (nt 2304–2324, 5′‐TTGATAAGATAGGGGCATTTG‐3′), with 5 min hot start followed by 30 cycles of 94^°^C for 30 sec, 50^°^C for 30 sec, and 72^°^C for 90 sec. Second round PCR was carried out on 5 µl of the first round product in a 50 µl reaction using primers CPRF1 (nt 1678–1695, 5′‐CAATGTCAACGACCGACC‐3′) and CPRR1 (nt 1928–1948,

5′‐GAGTAACTCCACAGTAGCTCC‐3′) and 5 min hot start, then 30 cycles of 94^°^C for 30 sec, 55^°^C for 30 sec, and 72^°^C for 30 sec.

HBV DNA positive products were sent to The Sangon Biotech (Shanghai, China) for sequencing using a BigDye Terminator V3.1 Cycle Sequencing kit (Applied Biosystems, Foster City, CA) with sequencing primer LSBI1 and HCO2 for the preS/S sequence and CPRF1for the BCP/precore sequence.

### Measurement of Viral Loads

Serum HBV DNA concentrations were quantified by real time PCR using commercial reagents (Shanghai ZJ Bio‐Tech Co., Ltd. (Shanghai, China)) in an ABI Prism 7500 sequence detection system (Applied Biosystems) with a dynamic range of 24.4–10^9^ IU/ml, using HBV primers and a dual labeled TaqMan probe, as described previously [Fang et al., 2009].

### HBV Genotyping

HBV genotypes were determined using the sequences of the S ORF and the STAR program [http://www.vgb.ucl.ac.uk/starn.shtml] [Myers et al., 2006] and the NCBI Genotyping Tool (http://www.ncbi.nlm.nih.gov/projects/genotyping/formpage.cgi).

### Statistical Analysis

The data are presented as median (range) and as numbers with percentages as appropriate, unless otherwise stated. Continuous and categorical variables were compared between groups using, respectively, the Mann‐Whitney test and the chi‐square/Fisher's exact test. Pearson's correlation coefficient (r) was used to describe the correlation between two continuous, normally distributed variables. Spearman's correlation was used where variables were not distributed normally. Simple and multiple linear regression analyses were performed to identify independent factors for serum HBsAg titer quantification. For the regression analyses, a logarithmic transformation was applied to all viral load and HBsAg measurements prior to analysis to achieve an approximately normal distribution. All *P*‐values were two‐tailed and *P* < 0.05 was considered to be significant. All statistical analyses were performed using the SPSS software (ver.16.0; Chicago, IL).

## RESULTS

### Baseline Characteristics

**Figure 1:**
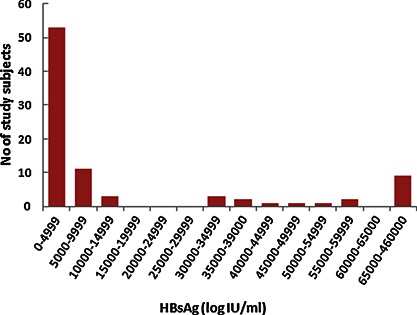
Distribution of serum HBsAg titers. The number of individuals in different ranges of HBsAg titer. The distribution of serum HBsAg titer across the study subjects is skewed.

Eighty‐nine study subjects were recruited from the Long An cohort, 52 males and 37 females. The mean ages were 37.5 ± 6.2 years. Twenty four (27%) study subjects were HBeAg‐positive and 65 (73%) were HBeAg‐negative. There are 23 samples with one of following mutations, PreS deletions or preS2 initiation codon mutation, BCP double mutations (T^1762^A^1764^), and preC stop mutation (nt1896), 24 samples with two of these mutations, and five samples with all of these mutations. In additional, there are 17 samples without any of these mutations. Therefore, there are 52 samples with mutated sequences. Overall, 25.8% of the study subjects had abnormal ALT levels (>40 IU/ml). No stop mutation in the S gene or amino acids changes in the “a” determinant of HBsAg was found. Three genotypes were identified, B, C, and I, with a prevalence of 64%, 30.3%, and 5.6% respectively. The distribution of serum HBsAg titer across the study subjects was skewed (Fig.1); most (61.6%) had HBsAg titers below 5000 IU/ml. The median (range) of HBsAg is 2.9 × 10^3^ (IU/ml) (5.0 × 10^1^–4.6 × 10^5^). The median (range) of viral loads is 3.0 × 10^5^ (IU/ml) (5 × 10^2^–4.8 × 10^8^) (Table I).

**Table I:** Characteristics of Subjects According to the Status of HBeAg

Characteristics	All subjects	HBeAg (+)	HBeAg (−)	*P*
N	89	24	65	
Age, years, mean (SD^a^)	37.5 (6.2)	36.2 (5.8)	37.9 (6.3)	0.220
Male sex, n (%)	52 (58.4)	16 (18)	36 (40.4)	0.338
HBV genotypes, n (%)				0.679
B	57 (64)	16 (66.7)	41 (63.1)	
C	27 (30.3)	6 (25.0)	21 (32.3)	
I	5 (5.6)	2 (8.3)	3 (4.6)	
HBV DNA,I U/ml, median (range)	3.0 × 10^5^ (5 × 10^2^‐4.8 × 10^8^)	1.5 × 10^8^ (1.9 × 10^6^‐4.8 × 10^8^)	7.6 × 10^4^ (5 × 10^2^‐5.9 × 10^7^)	0.000
HBsAg, IU/ml, median (range)	2.9 × 10^3^ (5.0 × 10^1^‐4.6 × 10^5^)	4.5 × 10^4^ (7.6 × 10^2^‐4.6 × 10^5^)	2.4 × 10^3^ (5 × 10^1^‐5.6 × 10^4^)	0.000
Abnormal ALT^b^, n (%)	23 (25.8)	5 (5.6)	18 (20.2)	0.512
T^1762^A^1764^ mutations, n (%)	38 (42.7)	3 (3.4)	35 (39.3)	0.000
PreS deletion or PreS2 start codon mutations	27 (30.3)	5 (5.6)	22 (24.7)	0.236
PreC stop mutation, n (%)	32 (46.4, 32/69)	0	32 (46.4, 32/37)	0.000

^a^ SD, standard deviation; ALT.

^b^ Alanine aminotransferase.

### Distribution of Serum HBV DNA Concentrations Between Wild Type and Mutated HBV Sequences

The viral loads of individuals with wild type sequences in the PreC and BCP regions are significantly higher than of those with mutations in these regions. Data from the follow up samples were consistent with this finding. Although the difference in viral loads between the PreS/S wild type group and mutation group in the first and third round samples is not significant, that in the second round is on the border of significance (Table II). These results suggest that, in most cases, viral loads of mutant HBV are lower than those of wild type HBV.

**Table II:** Distribution of Serum HBV DNA Levels Between Wild Type and Mutated Sequences of HBV

	First round		Second round		Third round	
Sequence status	Median IU/ml	*P‐*value	Median IU/ml	*P‐*value	Median IU/ml	*P‐*value
PreS/S wild type	2.095 × 10^5^	0.675	2.455 × 10^5^	0.047	2.16 × 10^5^	0.328
PreS/S mutant	5.51 × 10^5^		9.01 × 10^3^		2.355 × 10^5^	
BCP wild type	5.36 × 10^6^	0.002	5.52 × 10^5^	0.011	8.1 x 10^5^	0.01
BCP mutant	1.009 × 10^5^		1.86 × 10^4^		9.045 × 10^4^	
PreC wild type	9.0 × 10^6^	0.001	2.56 × 10^6^	0.001	4.58 × 10^6^	0.001
PreC mutant	7.215 × 10^4^		5.1 × 10^3^		2.255 × 10^4^	

### The Correlation Between HBsAg Titers and Serum HBV DNA Concentrations According to the HBeAg Status

**Figure 2:**
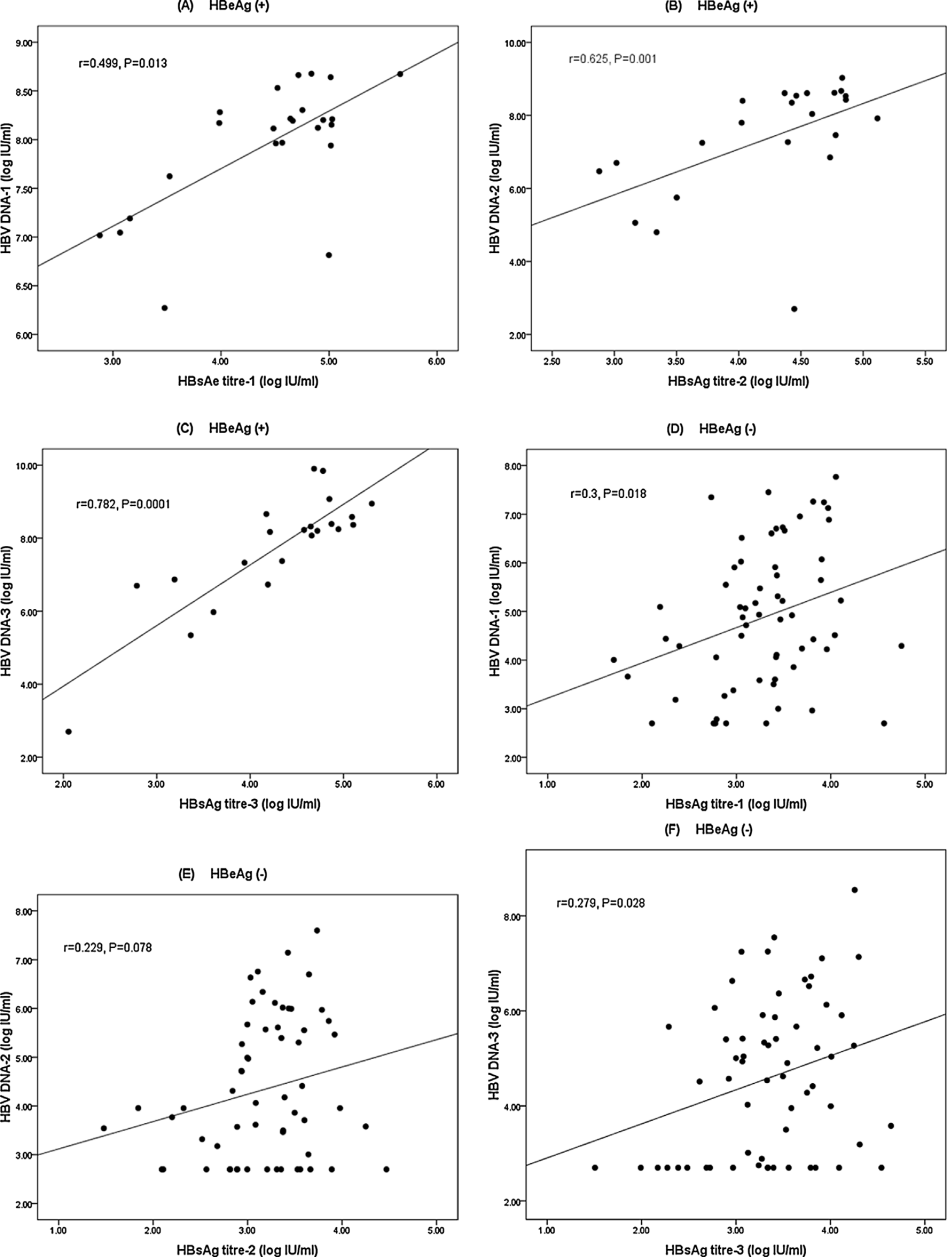
The correlation between serum HBsAg titers and HBV DNA concentrations according to the HBeAg status. Correlation between serum HBsAg titers and HBV DNA concentrations in HBeAg positive individuals in the first (A), second (B) and third (C) round samples and in HBeAg negative individuals in the first (D), second (E) and third (F) round samples.

In the first round of follow up, the serum HBsAg titer in total was positively correlated with serum HBV DNA (r = 0.642, *P* < 0.001) and this positive correlation was seen in both HBeAg positive and negative subjects (r = 0.449, *P* = 0.013; r = 0.300, *P* = 0.018, respectively) (Fig. 2A and D). The correlation could also be seen in the third round samples (r = 0.782, *P* < 0.001for HBeAg positive group; r = 0.279, *P* = 0.028 for the HBeAg negative group, respectively) (Fig. 2C and F). In the second round samples, the correlation was only seen in the HBeAg positive group (Fig. 2B) and not in HBeAg negative group (r = 0.229, *P* = 0.078) (Fig. 2E). However, the *P‐*value is close to significance. These findings suggest that the correlation between serum HBsAg levels and viral loads may not differ between HBeAg positive and negative individuals.

### The Correlation Between HBsAg Titers and Serum HBV DNA Concentrations According to Phases of Chronic HBV Infection

Chronic HBV infection has a complicated course and four phases have been described: immune tolerant, immune clearance, inactive and immune escape phase [Brooks et al., 2013]. The correlation between serum HBsAg titers and HBV DNA concentrations was examined for each phase. No correlation was found in any of the four phases (n = 19, r = 0.423, *P* = 0.071; n = 5, r = 0.838, *P* = 0.077; n = 45, r = 0.242, *P* = 0.110 and n = 17, r = 0.421, *P* = 0.092, respectively).

### The Correlation Between HBsAg Titers and Serum HBV DNA Concentrations According to PreS/S, BCP, and PreC Sequence Status

**Figure 3:**
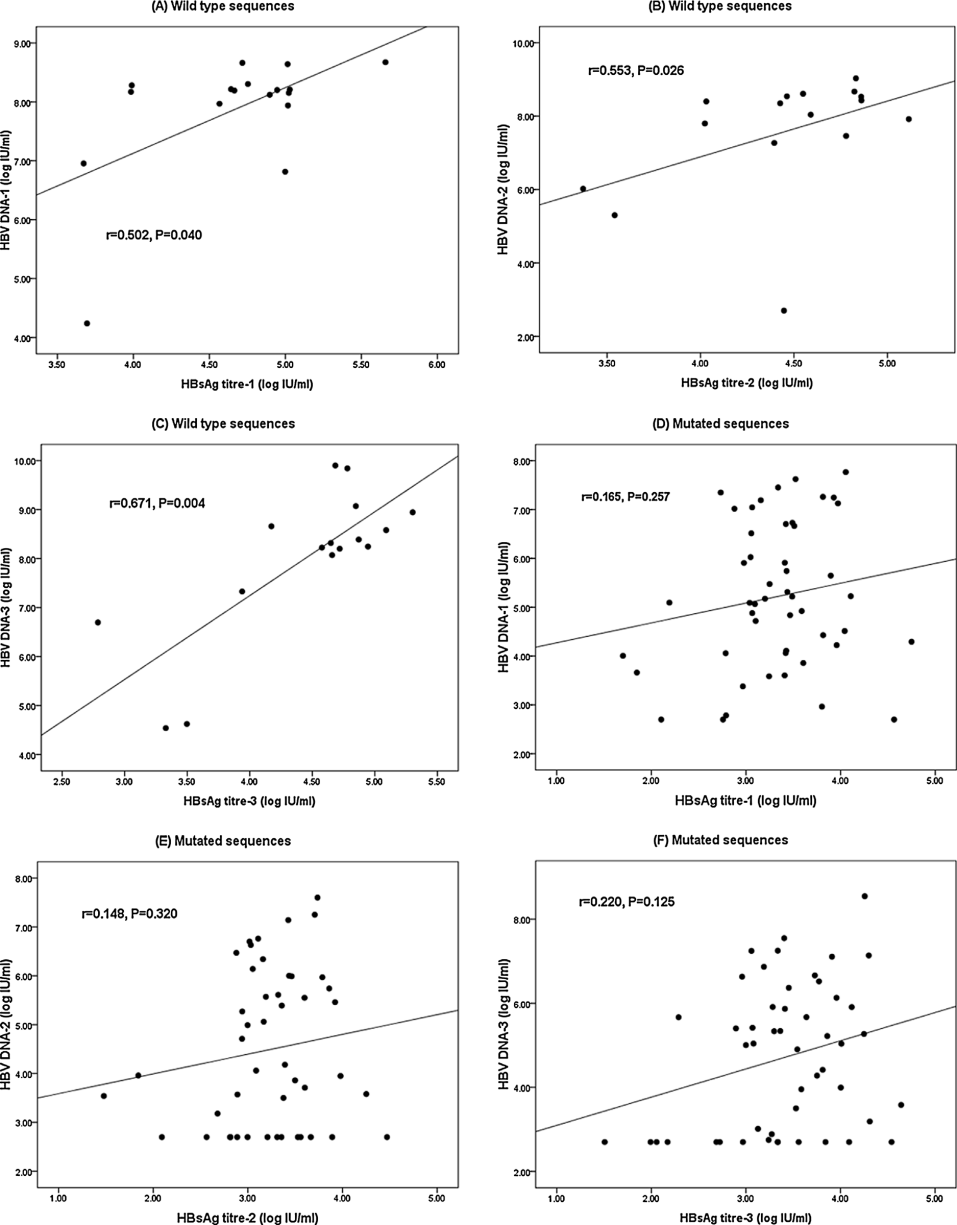
The correlation between HBsAg titers and serum HBV DNA concentrations according to the sequence status. Correlation between serum HBsAg titers and HBV DNA concentrations in individuals with wild type sequences in the first (A), second (B) and third (C) round samples and in those with mutated sequences in the first (D), second (E) and third (F) round samples. Mutations included preS deletions and mutations in the preS2 initiation codon, basal core promoter double mutations (T^1762^A^1764^) and preC stop mutation (nt1896).

The serum HBsAg titers were positively correlated with serum HBV DNA concentrations in the first round samples from the groups with wild type preS/S, BCP and PreC sequences (r = 0.502, *P* = 0.040) (Fig. 3A). However, the correlation was not seen in the first round samples from the groups with mutated sequences, including PreS deletions or preS2 initiation codon mutation, BCP double mutations (T^1762^A^1764^) and preC stop mutation (nt1896) (r = 0.165, *P* = 0.257) (Fig. 3D). In order to confirm these findings, data from second and third rounds of follow‐up were analyzed. Again, the serum HBsAg titers were also positively correlated with serum HBV DNA concentrations in the groups with wild type preS/S, PreC and BCP sequences (r = 0.553, *P* = 0.026; r = 0.671, *P* = 0.004, respectively) (Fig. 3B and C) but not in the groups with mutated sequences (r = 0.148, *P* = 320; r = 0.220, *P* = 0.125, respectively) (Fig. 3E and F). These findings suggest that the correlation between serum HBsAg levels and viral loads differs between individuals with wild type and mutated HBV sequences. Therefore, HBsAg quantitation could be used as a surrogate for viral load in wild‐type infections only.

It has been reported that there is a correlation between HBsAg and viremia levels in individuals infected with HBV with wild type preS/S sequences [Pollicino et al., 2012]. We carried out an analysis of those individuals with wild type preS/S sequences and also found the correlation in the first, second and third round samples (n = 59, r = 0.780, *P* < 0.001; n = 58, r = 0.715, *P* < 0.001; n = 58, r = 0.751, *P* < 0.001, respectively). Meanwhile, subgroups with BCP double mutations or PreC stop mutation were selected from those infected with wild type preS/S sequences for a stratification analysis. No correlation was found between HBsAg and viremia levels in the first, second and third round samples with BCP double mutations (n = 23, r = 0.362, *P* = 0.090; n = 23, r = 0.116, *P* = 0.599; n = 22, r = 0.172, *P* = 0.443, respectively) or PreC stop mutation (n = 16, r = 0.494, *P* = 0.052; n = 17, r = 0.420, *P* = 0.093; n = 18, r = 0.340, *P* = 0.167, respectively).

In order to eliminate the confounding effect of other mutations on the correlation between HBsAg and viremia levels, a further stratification analysis of individuals with BCP double mutations (T^1762^A^1764^) or preC stop mutation (nt1896) only was performed. Again, no correlation between serum HBsAg titer and viral loads was found in any round samples from those with BCP double mutations (n = 10,r=0.334, *P* = 0.346; n = 9, r = 0.202, *P* = 0.602; n = 10, r = 0.489, *P* = 0.151, respectively) or preC stop mutation (n = 9,r = 0.487, *P* = 0.193; n = 9, r = 0.517, *P* = 0.154; n = 10, r = 0.594, *P* = 0.070, respectively), suggesting that the correlation between serum HBsAg levels and viral loads varies according to the sequence status of preS/S, BCP, and preC.

### Analysis for Factors Associated With HBsAg Titers

Simple and multiple linear regression analyses were used to identify the factors that affect the titer of serum HBsAg. The independent variables included HBV DNA concentrations, HBeAg, gender, age, ALT, genotypes, BCP double mutations, PreC stop mutation, and PreS/S mutations (deletions and PreS2 initiation codon mutations). In the simple linear regression analysis, HBV DNA concentrations, HBeAg status, BCP double mutations, and PreC stop mutation were found to affect the titer of serum HBsAg. However, in the multiple linear regression analysis, sex, HBV DNA concentrations, and HBeAg status were found to affect significantly the levels of serum HBsAg. HBV sequence mutations (PreS/S mutations, BCP and PreC) did not affect the serum HBsAg titer (Table III).

**Table III:** Simple and Multiple Linear Regression Analysis of Factors Associated With Serum HBsAg Levels

	Variables	Unstandardized Coefficients		Standardized Coefficients	t	*P*
		B	Std. Error	Beta		
Simple analysis	DNA	0.278	0.036	0.642	7.668	0.000
	HBeAg	1.148	0.154	0.631	7.464	0.000
	Sex	−0.120	0.179	−0.073	−0.672	0.503
	Age	−0.024	0.014	−0.180	−1.673	0.098
	PreS/S	−0.175	0.191	−0.100	0.919	0.361
	BCP	−0.492	0.172	−0.297	−2.856	0.005
	PreC	−0.635	0.191	−0.383	−3.316	0.002
	Genotypes	−0.040	0.185	−0.024	−0.216	0.829
	ALT	0.000	0.001	−0.026	−0.238	0.813
Multiple analysis	HBeAg	0.752	0.251	0.413	2.999	0.004
	Sex	−0.393	0.157	−0.237	−2.501	0.015
	DNA	0.145	0.063	0.316	2.311	0.024

## DISCUSSION

The principal findings of this study are that the correlation between serum HBsAg levels and viral loads varies according to wild type and mutated HBV sequences. The correlation between serum HBsAg levels and viral loads may not differ between HBeAg positive and negative individuals or among the phases of chronic HBV infection. Viral loads of mutant HBV are usually lower than those of the wild type. HBV DNA concentrations and HBeAg status significantly affect the levels of serum HBsAg. The strength of this study is that the results of cross‐sectional analysis are supported by the results of longitudinal analysis, which overcomes the deficiencies associated with fluctuations of HBsAg. A weakness of the study is that the results were obtained for genotypes B and C and it is unclear whether they hold true for other genotypes.

There have been many studies of the correlation between serum HBsAg titer and HBV DNA but, unfortunately, the results are contradictory [Wiegand et al., 2008; Su et al., 2010; Ganji et al., 2011; Sun et al., 2012; Suh et al., 2014]. Although some studies suggested that the correlation between quantitative HBsAg titers and serum HBV DNA concentrations differs between patients with HBeAg‐positive and HBeAg‐negative CHB [Thompson et al., 2010], this finding was not confirmed by others [Su et al., 2010; Sun et al., 2012; Seto et al., 2012; Alghamdi et al., 2013; Suh et al., 2014]. The studies all involved cross‐sectional analysis. A single measurement may not be ideal for evaluating the correlations between serum HBsAg titers and HBV DNA concentrations because both may fluctuate over time [Chu et al., 2002; Su et al., 2013]. Recently, a study showed that the correlation between serum HBsAg titers and HBV DNA concentrations differs between individuals with wild type and mutated preS/S sequences. However, this study also was cross‐sectional and it did not address the correlation according to sequence status of the BCP and preC region [Pollicino et al., 2012]. In this study, we found that the correlation between serum HBsAg titers and HBV DNA concentrations differs not only between individuals with wild type and mutated preS/S sequences, but also between individuals with wild type and mutated BCP/preC sequences. Furthermore, these findings were confirmed by longitudinal analysis and, therefore, the results are reliable.

HBsAg is one of the products of HBV replication [Locarnini and Bowden, 2012]. Serum HBsAg titers should correlate with HBV DNA levels. However, it is possible for the association to be broken because the inhibition of HBV DNA replication and HBV gene expression occurs through independent mechanisms [Guidotti et al., 1996]. It has been found that HBV DNA declined rapidly during entecavir treatment while the decline of serum HBsAg titer was slow [Seto et al., 2014]. Most HBV mutations are subject to selection forces from host immune surveillance, antiviral therapy and replication fitness [Xu et al., 2013]. Therefore, the appearance of mutations means that HBV replication has encountered immune responses, which may influence the replication. In this study, the viral loads of the mutant groups are significantly lower than those of the wild type groups. Therefore, it is possible that the association between HBsAg production and HBV replication breaks down in this case. If so, it is not difficult to understand that there is no correlation between serum HBsAg titer and HBV DNA concentrations in the mutant groups.

Because of low cost and simplicity, many investigators have tried to use HBsAg as a surrogate marker of serum HBV DNA in chronic HBV infection to predict the outcome of the infection and the response to antiviral therapy. However, this approach has not been used in clinical practice because it has yielded contradicting results [Kuhns et al., 2004; Ganji et al., 2011; Su et al., 2010; Sun et al., 2012]. Our findings suggest that HBsAg quantitation could be used as a surrogate for viral load in wild‐type infections only. Therefore, the use of HBsAg titer as a surrogate marker of serum HBV DNA has to take into account the sequence status of the BCP and PreC regions but not necessarily of PreS/S, which is important to guide the use of HBsAg as a surrogate for viral load. Considering the cost of sequencing, HBsAg titration would be better not recommended as a surrogate for viral load quantification in regions where HBV genome sequencing is not easily accessible.

The correlation between serum HBsAg titres and HBV DNA concentrations between HBeAg positive and negative individuals remains uncertain. Some studies found this correlation in HBeAg‐positive CHB [Su et al., 2010; Sun et al., 2012; Suh et al., 2014] while others found it in HBeAg‐negative CHB [Chu et al., 2002; Alghamdi et al., 2013]. The correlation between serum HBsAg and HBV DNA concentrations could be seen in both HBeAg‐positive and negative patients [Jang et al., 2013]. We obtained similar results in this study. However, in our study no correlation was found between serum HBsAg titer and HBV DNA concentration in cases of the preC termination mutation known to prevent the synthesis of HBeAg. The association could be seen in only those patients with wild type preC (data not shown). This finding may help explain why some studies found the correlation in HBeAg‐negative individuals while others did not.

Although various studies found that the HBsAg titers correlated with HBV DNA in some phases of chronic HBV infection [Antaki et al., 2012; Suh et al., 2014], no such correlation was found in the four phases in this study. Some studies suggested that age and HBV DNA are the important parameters for HBsAg [Jang et al., 2011; Matsumoto et al., 2012]. However, we found in this study that age is not associated with the titer of HBsAg.

One of the important aims of HBsAg quantification is to predict treatment outcome [Martinot‐Peignoux et al., 2013]. Our data here were obtained from treatment‐naïve, asymptomatic carriers. We do not know whether our findings may be replicated in those undergoing antiviral therapy. In the future, we plan to determine whether there is a correlation between serum HBsAg and viral loads in patients undergoing antiviral therapy.
